# Transesophageal echocardiography (TEE) in cardiac arrest: results of a hands-on training for a simplified TEE protocol

**DOI:** 10.1186/s13089-020-00189-0

**Published:** 2020-08-18

**Authors:** Peiman Nazerian, Giuliano De Stefano, Giovanni Albano, Vera Gaspari, Sergio Bevilacqua, Valter Campagnolo, Pierluigi Stefàno, Stefano Grifoni

**Affiliations:** 1 Department of Emergency Medicine, Careggi University Hospital, Largo Brambilla 3, 50134 Florence, Firenze, Italy; 2 Department of Anaesthesiology, Careggi University Hospital, Florence, Italy; 3 Department of Cardiac Surgery, Careggi University Hospital, Florence, Italy

**Keywords:** Transesophageal echocardiography, Cardiocirculatory arrest, Emergency medicine, Emergency department, Resuscitation, Ultrasound, Training course

## Abstract

**Background:**

Integration of transesophageal echocardiography (TEE) with Focused Cardiac Ultrasound (FoCUS) can impact decision-making, assist in the diagnosis of reversible etiologies and help guiding resuscitation of patients with cardiac arrest.

**Objective:**

To evaluate the ability of emergency physicians (EPs) to obtain and maintain skills in performing resusTEE after a course with clinical training in the cardiac surgery theatre.

**Methods:**

Ten EPs without previous TEE experience underwent a resusTEE course, based on a 2-h workshop and an 8-h hands-on training. The training was performed in a cardiac surgery theatre tutored by cardiovascular anesthesiologists. The six taught views were mid-esophageal four-chamber (ME4CH), mid-esophageal long axis (MELAX), mid-esophageal two-chamber (ME2CH), mid-esophageal bicaval view (MEbicaval), transgastric short axis (TGSAX) and aorta view (AOview). The EPs were evaluated by a cardiovascular anesthesiologist at the end of the course as well as after 12 weeks according to a standardized evaluation method. Once the course was completed, resusTEE exams, performed by EPs in Emergency Department (ED), were monitored for a 12-week period.

**Results:**

The average assessment of the ten EPs by the tutors was higher than 4 points out of 5, both at the end of the course and after 12 weeks. Probe insertion, acquisition and interpretation of the different views scored on average more than 4 points out of 5 except for TGSAX that showed worsening after 12 weeks. Trainees performed twelve resusTEE exams in ED in patients with out-of-hospital cardiac arrest (OHCA) over 12 weeks after the course. EPs used only four out of six taught views in clinical practice, in the following order of frequency: ME4CH, AOview, MEbicaval and MELAX.

**Conclusions:**

EPs, after a course with clinical training in the cardiac surgery theatre, can successfully acquire and maintain the skills needed to perform resusTEE. However, among the six views learned in the course, EPs used only four of them (ME4CH, MEbicaval, MELAX and AOview).

## Background

Emergency physicians (EPs) routinely use Focused Cardiac Ultrasound (FoCUS) in the management of cardiac arrest. The European Resuscitation Council guidelines suggest integrating FoCUS into the advanced life support algorithm for the identification of potentially reversible causes of cardiac arrest, pseudo-pulseless electrical activity (PEA), and to help the decision to interrupt cardiopulmonary resuscitation (CPR) [1]. However, an important limitation of FoCUS is the technical difficulty to obtain adequate cardiac windows in the few seconds available during the scheduled pauses in compressions performed for rhythm check. Common reasons for the poor acoustic windows obtained in cardiac arrest patients are insufflation of air into the stomach during pulmonary ventilation, and the presence of defibrillator pads, automated compression devices, and other monitoring equipment [2]. The aforementioned limitations of FoCUS could be overcome by adding other ultrasound views with transesophageal echocardiography (TEE) [3].

TEE offers several advantages in critical care because of the excellent acoustic window, due to the position of the TEE probe in the esophagus or stomach, very close to the heart [4, 5]. Additionally, TEE provides a better identification of aortic dissection [6] and can be used to guide cannulation for extracorporeal life support (ECLS) [7]. Furthermore, it can be performed during CPR without interrupting chest compressions, offering continuous support to improve CPR quality and to decide when to discontinue resuscitation [3, 8–10].

Although, only small, single-center observational studies have been published on the use of focus TEE in the ED [3, 11]. The American College of Emergency Physicians recommends the utilization of a simplified protocol of TEE (resuscitative TEE, resusTEE) including only few of the twenty-eight views needed for a comprehensive TEE examination [12, 13]. The best simplified institutional protocol of resus TEE and the methods of training needed for EPs to learn the technique should be defined before implementing the use of TEE in the ED.

TEE is rarely used in ED and it is performed in emergency situations, when teaching is often impossible; for these reasons, practical skills need to be learned outside ED. High-fidelity simulators have an important and well-known role in the learning of new skills and are currently used also for resusTEE training. Arntfield et al. evaluated the ability of EPs to acquire and maintain the skills required for performing TEE at 6 weeks after a course based on simulations [14]. However, TEE simulators have elevated costs and are available only in a few EDs. Clinical training performed in a “controlled” environment such as the cardiac surgery theatre could be an alternative option that still needs to be evaluated. In our institution, we have no simulator available for training. Therefore, we decided to implement resusTEE by training EPs in the cardiac surgery theatre.

The primary objective of this study was to evaluate the ability of EPs to acquire and maintain the skills to perform six views of resusTEE after a 10-h course based on a clinical training. As additional endpoint, we evaluated which views were used in clinical practice in ED, during the period of implementation of the technique.

## Methods

### ResusTEE course and protocol

The study was approved by the Regional Ethical Committee.

ResusTEE course was attended by ten EPs (six specialists and four residents) with at least 1 year of experience in FoCUS. The course was composed of 2 hours of formal lectures and 8 hours of a hands-on clinical training. The lectures included the review of basic TEE principles, indications and contraindications for TEE, demonstration of probe insertion and the technique needed to acquire six views of a simplified TEE protocol and to identify, through video loops, anatomic structures as well as the most common pathologic findings.

The clinical training was performed in three cardiac surgery operating rooms with cardiovascular anesthesiologists, certified as tutors in adult transesophageal echocardiography by the European Association of Cardiovascular Imaging. Each operating room was equipped with an ultrasound system provided with a 7.0-MHz TEE probe: SONOS 5500 (Phillips Medical Systems, Bothell, WA), iE33 (Philips, Andover, MA) and EPIQ 7(Philips, Andover, MA).

The tutor guided the trainees to acquire the manual and interpretive skills needed for the insertion of the probe, acquisition, and interpretation of six different TEE views. The trainees inserted the probe in patients scheduled for elective cardiac surgery, already intubated, under general anesthesia. TEE is routinely performed in such patients for monitoring purposes and to check the result of surgery. Therefore, patients were not subjected to any additional risk due to the teaching procedure and, since the same TEE views were used also for clinical purposes, the training did not interfere with the surgery.

**Fig. 1:**
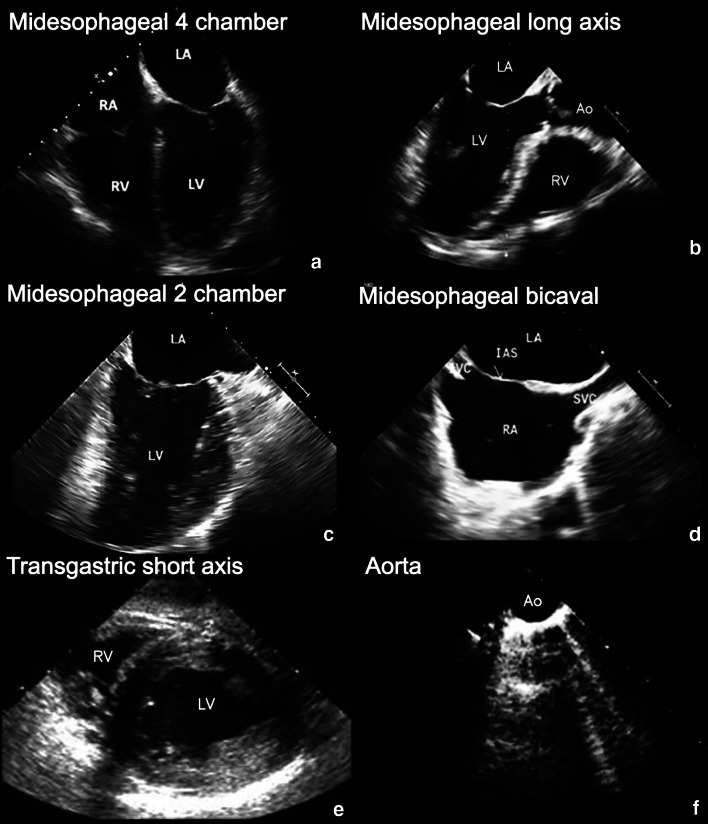
**a** Mid-esophageal four-chamber view (ME4CH)—useful for evaluation of right and left ventricle function and size, mitral and tricuspid valve alterations, pericardial effusion and, during a pulse check, for the assessment of the presence of a perfusing rhythm. **b** Mid-esophageal long-axis view (MELAX)—helpful for the evaluation of the left ventricular systolic function and, during CPR, for the evaluation of compression adequacy and location. **c** Mid-esophageal two-chamber view (ME2CH)—valuable for the evaluation of the left ventricular systolic function and the regional motion of the anterior and inferior wall. **d** Mid-esophageal bicaval view (MEbicaval)—allows the evaluation of superior vena cava thrombus, catheters or venous cannula position (as for ECLS) and respiratory dimensions variations that can be related to volume status and fluid responsiveness. **e** Transgastric short-axis view (TGSAX)—provides information about pericardial effusion, left ventricle systolic function and regional wall motion abnormalities. **f** Aorta view (AOview)—useful for guiding vessel cannulation for ECLS and for the identification of aortic dissection/aneurysm involving the aortic arch and the descending thoracic aorta. *Ao* aorta, *IAS* interatrial septum, *IVC* inferior vena cava, *LA* left atrium, *LV* left ventricle, *RA* right atrium, *RV* right ventricle, *SVC* superior vena cava

ResusTEE protocol was composed of six sequential views: mid-esophageal four-chamber (ME4CH) (Fig. 1a–Additional file 1: video 1a), mid-esophageal long axis (MELAX) (Fig. 1b–Additional file 2: video 1b), mid-esophageal two-chamber (ME2CH) (Fig. 1c–Additional file 3: video 1c), mid-esophageal bicaval view (MEbicaval) (Fig. 1d–Additional file 4: video 1d), transgastric short axis (TGSAX) (Fig. 1e–Additional file 5: video 1e) and aorta view (AOview) (Fig. 1f–Additional file 6: video 1f) (Additional file). This protocol differed from the one proposed by the American College of Emergency Physicians because of the addition of three views (ME2CH, MEbicaval and AOview) [12] and from the protocol proposed by the University of Pennsylvania due to the addition of two views (ME2CH and AOview) [15].

### Skill assessment throughout the course

During the practical training, the trainees were monitored and the effective hours in which they handled the probe, the number of exams performed, and the type and number of inspected pathological findings were registered. Furthermore, at the end of the training period, an observer medical student registered the time needed to insert the probe and to acquire the six views of the protocol. One of the two anesthesiologists rated the skills of the trainees. The same assessment was repeated after 2 h of hands-on retraining, 12 weeks after the completion of the course, to evaluate skills retention over time.

The skill assessment consisted of a practical exam, by which the technique of probe insertion, acquisition and interpretation of the six different views (ME4CH, ME2CH, MELAX, MEbicaval, TGSAX, AOview) were evaluated and rated separately. The tutors used a rating system (from 1 to 5) as follows: 1 = inadequate, 2 = insufficient, 3 = sufficient, 4 = good and 5 = excellent.

The insertion of the probe was assessed considering the quickness time required for insertion and taking into account the possible need for help from the tutor, according to the following scale: 1 = insertion failed; 2 = insertion achieved only with substantial tutor’s intervention; 3 = insertion achieved in > 2 min; 4 = insertion achieved in > 1 and ≤ 2 min; 5 = insertion achieved in ≤ 1 min. The learner’s ability to acquire each of the 6 views was also assessed on the basis of a similar evaluation scale previously published taking into account quickness the time required to obtain the view and the amount of help needed by the learner to reach the target [16]. This scale has been adapted on the basis of the aforementioned 5 skill levels according to the following scheme: 1 = image acquisition failed; 2 = image achieved only after substantial tutor’s help; 3 = image achieved slowly but with small tutor instruction; 4 = image achieved slowly but without any tutor instruction; 5 = image obtained quickly and fluidly as part of a complete examination without any help from the tutor. To attribute the score, the tutor also took into account the objective difficulties encountered both by himself and by the learner, in clinical cases in which the acoustic window was suboptimal. Therefore, in these cases, a view was considered as adequately achieved if similar to the best obtainable by the certified tutor. Similarly, criteria to consider an adequately interpreted view (rating  ≥ 3) were the confidence in the correct identification of all the anatomic structures and the completeness in the discussion of all the potential pathological conditions that could be identified by each view. In this regard, the tutor assigned a score ranking from 1 to 5 according to his personal experience and at his discretion.

### Implementation of resusTEE performed by EPs in clinical practice

For 12 weeks, after the end of the course, we monitored the effective application of resusTEE performed by all the trainees in ED. Each one of the EPs trained in the course was involved in resuscitation in case of out-of-hospital cardiac arrest (OHCA) and the decision to perform a resusTEE in addition to FoCUS was decided by the resuscitation team leader. The FoCUS was already part of the standard management of OHCA in our ED and was always performed according to the European Resuscitation Council guidelines [1]. ResusTEE was performed by EPs, with the supervision of a cardiologist or an anesthesiologist, part of the resuscitation team, after patients’ intubation, using a dedicated ultrasound machine (Vivid S5 ultrasound multiprobe machine provided with a GE 6Tc-RS TEE Probe, GE Healthcare, Wauwatosa, WI, USA), available in the shock room 24 h a day, 7 days a week. The time from the patient’s arrival in the shock room to the successful probe insertion, the time needed to complete the TEE exam and the different views performed by EPs were recorded by a medical student. The resuscitation team leader completed a standardized form after having finished the clinical management. The form reported if resusTEE had led to a modification of the diagnosis made by FoCUS, when it suggested a shift of the site of chest compression during CPR, when it was used to guide vessel cannulations as well as for the interruption of CPR.

The results were presented as a percentage for dichotomous variables, as mean ± standard deviation for normally distributed variables. The analysis was conducted using Excel 15.30 (Office, Microsoft, United States of America).

## Results

The ten trainees had 8.3 ± 6.2 years of experience in FoCUS, but none had any previous TEE experience.

All trainees participated in all the courses and each trainee spent 8.5 ± 3 h in the operating room, 6.8 ± 3 h effectively handling the probe and performed TEE in an average of 9.8 ± 2.9 patients. At the end of the course, the average time needed for probe insertion was 75 ± 42 s, and 4.2 ± 3 min to complete the 6 views of the protocol.

The overall result of the exam was rated by the tutors as “good to excellent” both at the end of the course (mean rating 4.8 ± 0.4) and after 12 weeks (mean rating 4.8 ± 0.6). The evaluation of the skill for the insertion of the probe, the acquisition and interpretation of the six views was rated by the tutors as “good to excellent” except for the transgastric short axis, that showed worsening of the acquisition ability after 12 weeks (Table 1).

**Table 1:** Probe insertion, acquisition, and interpretation of resusTEE views at the end of the course and after 12 weeks rated by tutors

Tutor’s assessment	End of the course Mean ± SD	After 12 weeks Mean ± SD	Difference mean ± SD
Probe insertion	4.8 ± 0.4	4.7 ± 0.4	0.1 ± 0
*Views acquisition*			
Mid-esophageal four-chamber	4.8 ± 0.4	4.8 ± 0.6	0 ± 0.2
Mid-esophageal two-chamber	4.8 ± 0.4	4.9 ± 0.3	0.1 ± 0.1
Mid-esophageal long axis	4.8 ± 0.4	4.9 ± 0.3	0.1 ± 0.1
Mid-esophageal Bicaval view	4.7 ± 0,4	4.8 ± 0.6	0.1 ± 0.2
Aorta and aortic arch views	4.8 ± 0.4	4.8 ± 0.6	0 ± 0.2
Transgastric short axis	4.5 ± 0.7	3.7 ± 1.2	-0.8 ± 0.5
Color doppler images	4.8 ± 0.4	5	0.2 ± 0.4
*Views interpretation*			
Mid-esophageal four-chamber	4.8 ± 0.4	4.8 ± 0.6	0 ± 0.2
Mid-esophageal two-chamber	4.8 ± 0.4	4.9 ± 0.3	0.1 ± 0.1
Mid-esophageal long axis	4.8 ± 0.4	4.9 ± 0.3	0.1 ± 0.1
Mid-esophageal Bicaval view	4.8 ± 0.4	4.8 ± 0.6	0 ± 0.2
Aorta and aortic arch views	4.8 ± 0.4	4.8 ± 0.6	0 ± 0.2
Transgastric short axis	4.7 ± 0.4	4.2 ± 1.3	− 0.5 ± 0.9
*Overall*	4.8 ± 0.4	4.8 ± 0.6	0 ± 0.2

*SD* standard deviation

Table 2 summarizes the number of different operative findings evaluated by trainees during the course in the cardiac surgery theatre.

**Table 2:** ResusTEE operative findings during the hands-on training

ResusTEE operative findings	Frequency (number of occurrences)	Mean for each trainee ± SD
Reduced global left ventricular function	66	6.6 ± 4.8
Regional wall motion abnormalities	64	6.4 ± 4.9
Guidance of insertion of catheters	64	6.4 ± 4.9
Absence of cardiac motion	44	4.4 ± 3.2
Right ventricular dysfunction	26	2.6 ± 1.8
Pericardial effusion/tamponade	24	2.4 ± 1.7

During the 12 weeks following the completion of the course, the ten trainees performed resusTEE in ED on twelve OHCA, intubated patients (1.2 ± 1.1 for each trainee). ResusTEE exams were performed on ten patients during RCP, while on the other two patients the exams were performed after the return of spontaneous circulation. In one patient with major thoracic trauma, although the probe was inserted, it was not possible to obtain any adequate view. Median time from the patient’s arrival in the shock room to the probe insertion was 14.4 ± 13.18 min and the time needed to complete resusTEE exam was 4.2 ± 1.4 min. The most used views in order of occurrence were ME4CH, AOview, MEbicaval and MELAX while ME2CH and TGSAX views were not used.

In nine patients (81%), resusTEE confirmed the diagnosis obtained by FoCUS (5 cases of acute coronary syndrome, 1 case of pulmonary embolism, 1 case of septic shock, 2 cases of undetermined cardiac arrest); while in 2 cases (19%), it led to changes in diagnosis and management: one case of acute aortic dissection previously diagnosed as acute coronary syndrome (Additional file 7: video 2) and one case of cardiac tamponade unidentified by FoCUS, because of a poor acoustic window. Furthermore, resusTEE was of pivotal importance during resuscitation in 4 cases, in which it indicated to shift the selected site of compression for cardiac massage over the left ventricle, showing appropriate opening of the mitral and aortic valves. Additionally, it guided vascular cannulation for ECLS in six patients (Additional file 8: video 3) and led to the interruption of CPR in other 4 cases.

## Discussion

Our study showed that EPs who attended a 2-h lectures and an 8-h hands-on clinical training in a cardiac surgery theatre can successfully acquire the skills needed to learn a simplified TEE protocol, and that these skills can be maintained after 12 weeks. A previous study on simulation training had showed similar results at the end of the course and after a shorter period (6 weeks) [14]. The perioperative environment is ideal to practice probe insertion and image acquisition because the patient is intubated under general anesthesia, and undergoes a long operation such as cardiac surgery. This is the reason why the time spent by the trainees effectively handing the probe was long in this study. Besides, trainees faced many different pathologic conditions and rapid changes in hemodynamics during the different phases of the cardiac surgical intervention. A potential disadvantage of this approach is the risk of mechanical injury from probe insertion and manipulation, given the employ of real patients for the training of clinicians who had no prior experience with this device, although we did not report any injury to patients during the training under the supervision of expert cardiovascular anesthesiologists. Another limitation is the lack of possibility of facing situations of cardiac arrest and CPR in the operating room, even if the trainees frequently observed the absence of cardiac motion. While resusTEE training based on simulators has already proved to be an optimal method, this study showed that clinical training could be a feasible alternative when simulators are not available [14, 15].

In the 12-week period after the course, the trainees performed resusTEE exams on few patients, corresponding to 22% of OHCA patients managed in the same period in ED. An excessively long median time from patient’s arrival in the shock room to the probe insertion was recorded. A further implementation in the practice of resusTEE is, therefore, mandatory. Since participants can maintain their abilities 12 weeks post-course but with some deterioration in their skills, and given the few opportunities to practice resusTEE in their clinical practice, it could be advisable to offer a continuous training with a 2-h retraining in the operating theatre every 12 weeks.

The trainees showed good performances in the acquisition and interpretation of all of the six suggested views, except for TGSAX view, for which a decrease of performance after 12 weeks was recorded. This view was not used in clinical practice in ED. Different factors could explain this finding: first of all, a technical reason, because TGSAX requires more complex movements to be obtained, and because the acquisition of the image may be prevented by anatomic factors, such as gastrectasia and hiatal hernia. Besides, some information such as evaluation of systolic function, wall motion abnormalities and presence of pericardial effusion can be derived from other easier views. However, we have to consider that TGSAX is an ideal view for anatomic monitoring when conducting resuscitation. It is less subject to cranio-caudal displacement, given the gastric location and it is the only view that characterizes all of the six major wall segments and allows even the inexperienced user to best evaluate wall motion and global LV function. Lastly, the TGSAX is the only view that teaches the value of the transgastric position and ante-flexion, thereby preparing the participants for the motor skills required to obtain additional views. Therefore, we believe that the importance of this view needs to be reinforced and more effort should be dedicated to its learning throughout the course.

The other view that was not used in clinical practice was ME2CH, despite being easily obtained. The reasons for the non-utilization of this view by the trainees are not technical, as for TGSAX, but only related to the few additive information achieved by this view during OHCA. Unlike TGSAX view, ME2CH was not included in the previous protocols and, thus, could be omitted from the protocol [12, 14, 15].

AOview was taught in our course, although not included in the previous resusTEE protocols [12, 14, 15]. In our study, this view was not only outstandingly acquired and interpreted during the course by trainees, but it was also frequently used in clinical practice in ED. This view was useful for guiding vessel cannulation for ECLS and as an additive view to MELAX for the diagnosis of acute aortic dissection. However, we must highlight that AOview was often used for guiding cannulations as our ED is a “hub” center for ECLS. This view would probably be less important and less commonly used in EDs without ECLS facilities.

Given the limited data (only a few cases performed on real patients), a further period of observation is mandatory to assess the inclusiveness of the AOview view in the protocol and its effective utility in emergency situations.

This study, as already reported by others [10], showed that EPs can easily learn resusTEE and that resusTEE is useful to make the correct diagnosis, take the decision to interrupt CPR, and be a guidance for vessel cannulation for ECLS. However, it is still unknown if resusTEE can modify the outcomes of critical patients.

## Limitations

All the participants were experts in FoCUS; therefore, it is not known if similar results can be obtained by EPs who have lower or no experience in FoCUS.

Practical training was carried out in one of the major Italian cardiac surgery centers with 3 operating theatres working simultaneously. This exposed trainees to many different cases, and this situation is uncommon in smaller centers.

Lastly, the utilization of resusTEE in clinical ED practice was monitored for a relatively short period of time and immediately after the end of the course, therefore no definitive conclusions can be drawn about which views should be included or excluded in a final and acknowledged simplified protocol; furthermore, the ten trainees performed on average just one study and their performances could only be assessed once.

## Conclusions

EPs can successfully learn and maintain the skills which are required for the use of a simplified resusTEE protocol through a 10-h theoretical–practical course, performed in a cardiac surgery theatre. While high-fidelity simulation is the ideal modality for the training of novices in TEE, clinical training in the operating theater can be a feasible alternative if simulators are not available. Among views incorporated in the previous resusTEE protocols, TGSAX was not used in clinical practice; therefore, its importance needs to be reinforced during the course.

## Supplementary information

**Additional file 1: Video 1a.** Mid-esophageal four-chamber view (ME4CH).

**Additional file 2: Video 1b.** Mid-esophageal long-axis view (MELAX).

**Additional file 3: Video 1c.** Mid-esophageal two-chamber view (ME2CH).

**Additional file 4: Video 1d.** Mid-esophageal bicaval view (MEbicaval).

**Additional file 5: Video 1e.** Transgastric short-axis view (TGSAX).

**Additional file 6: Video 1f.** Aorta view (AOview).

**Additional file 7: Video 2.** Acute aortic dissection.

**Additional file 8: Video 3.** Cannulation of vessels for ECLS.

**Additional file 9:** Standard technique used to obtain the six views of the simplified TEE protocol.

## Data Availability

The datasets used and/or analyzed during the current study are available from the corresponding author on reasonable request.

## Abbreviations

TEE: Transesophageal echocardiography
FoCUS: Focused cardiac ultrasound
OHCA: Out-of-hospital cardiac arrest
ResusTEE: Resuscitative TEE
EP: Emergency physician
ED: Emergency department
ECLS: Extracorporeal life support
PEA: Pulseless electrical activity
CPR: Cardiopulmonary resuscitation
SD: Standard deviation
