# Supplementary material for: Transesophageal echocardiography (TEE) in cardiac arrest: results of a hands-on training for a simplified TEE protocol
Source: Ultrasound J. 2020 Aug 18;12:41. doi: 10.1186/s13089-020-00189-0 (PMC7431479; doi:10.1186/s13089-020-00189-0)
Supplement: Supplementary file 9 — Additional file 9: Standard technique used to obtain the six views of the simplified TEE protocol. [file 13089_2020_189_MOESM9_ESM.docx]

**Standard technique used to obtain the six views of the simplified TEE protocol**

The mid esophageal four-chamber (ME4CH) was the first view to be obtained in the teaching algorithm. After the probe was advanced into the thoracic esophagus, the four heart chambers were visualized with the multiplane angle adjusted at 0 to 20 degrees. The second and third view were mid esophageal long axis (MELAX) view, obtained by increasing the multiplane angle to 110 - 160 degrees up and the mid esophageal two-chamber (ME2CH) view, obtained by increasing the multiplane angle to about 60 degrees up. The fourth view was the mid esophageal bicaval (ME bicaval), obtained by returning the probe to the ME4CH view, rotating the probe clockwise and reducing the multiplane to about 90 - 110 degrees. The fifth view, the transgastric short axis (TGSAX), was obtained by turning the multiplane angle to 0 degrees, advancing the probe, crossing the gastroesophageal junction to reach the stomach, and then anteflexing it to identify the left ventricle at the papillary muscle level. Finally, the aorta view (AOview) was obtained by rotating the probe counterclockwise from the ME4CH view, to identify the descending aorta - short axis. The transducer was then withdrawn and advanced to examine the entire descending thoracic aorta. The long axis was obtained with the multiplane at 90 degrees. At the level of the proximal descending aorta, the probe was withdrawn and then rotated clockwise to visualize the aortic arch.
